# Scientific Advancements in Composite Materials for Aircraft Applications: A Review

**DOI:** 10.3390/polym14225007

**Published:** 2022-11-18

**Authors:** Bisma Parveez, M. I. Kittur, Irfan Anjum Badruddin, Sarfaraz Kamangar, Mohamed Hussien, M. A. Umarfarooq

**Affiliations:** 1 Department of Manufacturing and Materials Engineering, Kulliyyah of Engineering, International Islamic University Malaysia, Kuala Lumpur 53100, Malaysia; 2 Centre of Advanced Materials, Faculty of Engineering, Universiti Malaya, Kuala Lumpur 50603, Malaysia; 3 Department of Mechanical Engineering, Faculty of Engineering, Universiti Malaya, Kuala Lumpur 50603, Malaysia; 4 Mechanical Engineering Department, College of Engineering, King Khalid University, Abha 61421, Saudi Arabia; 5 Department of Chemistry, Faculty of Science, King Khalid University, Abha 61413, Saudi Arabia; 6 Pesticide Formulation Department, Central Agricultural Pesticide Laboratory, Agricultural Research Center, Dokki, Giza 12618, Egypt; 7 Center of Excellence in Material Science, School of Mechanical Engineering, KLE Technological University, Hubballi 580031, India

**Keywords:** metal matrix composites, aircraft components, ceramic matrix composites, polymer matrix composites

## Abstract

Recent advances in aircraft materials and their manufacturing technologies have enabled progressive growth in innovative materials such as composites. Al-based, Mg-based, Ti-based alloys, ceramic-based, and polymer-based composites have been developed for the aerospace industry with outstanding properties. However, these materials still have some limitations such as insufficient mechanical properties, stress corrosion cracking, fretting wear, and corrosion. Subsequently, extensive studies have been conducted to develop aerospace materials that possess superior mechanical performance and are corrosion-resistant. Such materials can improve the performance as well as the life cycle cost. This review introduces the recent advancements in the development of composites for aircraft applications. Then it focuses on the studies conducted on composite materials developed for aircraft structures, followed by various fabrication techniques and then their applications in the aircraft industry. Finally, it summarizes the efforts made by the researchers so far and the challenges faced by them, followed by the future trends in aircraft materials.

## 1. Introduction

The accelerated growth in the modern aviation industry has led to advancements in aircraft materials. The primary motivators include cost reduction, weight reduction, and the extension of the service life of the components in the aircraft structures. The use of lightweight materials improves mechanical properties and fuel efficiency, flight range, and payload, as a result reducing the aircraft operating costs. Thus, researchers are working on the development of materials with optimized properties for weight reduction, fatigue resistance, corrosion resistance, and enhanced damage tolerance [1]. The proper selection of the material is crucial in designing the aircraft structure. Composite materials have been preferred extensively for the development of several military and commercial aircraft [2], as well as for Unmanned Aerial Vehicle (UAV) [3,4]. Over the last 80 years, Al-based alloys have dominated aerospace materials [5]. The high specific density, corrosion resistance, damage tolerance, and high-temperature resistance of Al alloys make them appealing for the manufacture of high-performance aircraft parts. Recent advances in the development of robust Al-Li and Al–Zn alloys, as well as the damage-resistant Al-Li and Al–Cu alloys, have resulted in enhanced fatigue and static strength, fracture toughness, and corrosion resistance by the virtue of variation in chemical composition and effective heat treatment [6,7,8,9]. Furthermore, Al metal matrix composites (MMC) are generally constituents of Al alloys (Al-Si, Al–Cu, Al-Si–Mg) as matrix materials reinforced with SiC, Al_2_O_3_, C, B, B_4_C, AlN, SiO_2,_ and BN mostly [10]. Al MMC possesses vital properties such as higher strength, significant wear-resistant, lower thermal expansion, and high specific modulus [11].

The magnesium sheets when used as a replacement for Al and steel exhibit greater potential for weight reduction depending on the stress profiles in various applications [12]. Although the density of magnesium is only a quarter of steel or two-thirds of Al, the tensile strength of 610 MPa can be achieved with Mg-based alloys [13]. Furthermore, Mg-based alloys have remarkable stiffness and damping capability. Due to significant improvements in the properties of Mg-based alloys, weight reduction and an increase in the payload for aircraft have been achieved [13,14]. However, the flammability and corrosive properties of Mg-based alloys limit their use in aircraft [15]. Titanium alloys possess substantially high strength in comparison to AL. However, based on the assumption that the component is not gage limited, the weight reduction can be attained by replacing aluminum despite being 60% high in density. At high temperatures, Titanium-based alloys, which include Ti-10V-2Fe-3Al, B120VCA, and Ti-6Al-4V, have a lower density and higher strength than high-strength steels. The main characteristics of various titanium alloys, as well as the production routes, were evaluated for their applications in the aerospace industry [16]. These metal matrix composites are typically strengthened by reinforcing boron, boron carbide, boron nitride, carbon, aluminum oxide, silicon carbide, silicon dioxide, and so on in the matrix [17]. Furthermore, ceramic matrix composites are capable of enduring high operating temperatures of 1400 °C [18] allowing them to meet the increasing demand for aircraft speed. Fiber-reinforced composites such as SiC–SiC and C–SiC are currently substituting the existing materials in crucial aerospace applications [19].

The development of fiber-reinforced polymer composite materials has resulted in significant advancement in the construction of lightweight structures [20]. Recently, the use of CFRP (Carbon Fiber Reinforced Plastic) in airframes and engine parts has increased to reduce aircraft fuel consumption. Carbon fiber-reinforced polymer (CFRP) has a minimum yield strength of 550 MPa, but its density is 1/5 of steel and 3/5 of Al-based alloys [21]. Although aerospace materials have made significant advances, there exist some significant challenges such as inadequate strength, which is insufficient to meet the increasing demand for lightweight materials. This review aims to discuss the composites developed for aircraft materials. The properties of the composites, their fabrication techniques, and their applications in various aircraft structures are also discussed. Finally, the challenges and the future scope in the development of aircraft materials are presented, followed by a summary.

## 2. Metal Matrix Composites

Generally, MMCs are classified based on their matrix material. Some of the most commonly used metal substrate configurations for aircraft applications are aluminum (Al)-based, magnesium-based, and titanium-based composites as presented in Table 1.

### 2.1. Aluminum-Based MMCs

Aluminum Matrix Composites (AMCs) are a sophisticated class of composite materials, wherein the Al or Al/Al alloys are reinforced with a secondary high-strength material, for instance, ceramics or fiber-reinforcements (carbon fibers). The properties such as strength, stiffness, and density of these materials can be tailored according to the applications where high performance is required. AMCs have higher strength and stiffness, can be operated at a higher temperature range, possess superior damage tolerance, better wear resistance, easier repairability, and can be recycled easily in comparison to unreinforced metals. AMCs offer as superior strength as steel with one-third of the weight.

Al alloys have been widely used for reducing weight, manufacturing, operating, and repairing costs for their structural applications in aircraft [1,22]. However, their usage in airframes is growing rapidly. This is evident in commercial aircrafts such as Airbus A350XWB, A380, and Boeing 787, also business aircrafts such as Dassault and Raytheon. The usage of these composites is increasing due to their improved performance as compared to conventional Al alloys. The composites not only reduce the weight but also affect maintenance costs [23]. AMCs can be used in harsh environments where reliability and safety are required, as they possess superior fatigue strength as compared to steel. AMCs find application in aircraft landing gear, high-pressure seals, and seats. AMCs meet the challenge of reducing the weight of landing gear significantly thereby allowing manufacturers to reduce the weight by as much as 30% as compared to conventional material [24].

It was observed that with the addition of SiC and Al_2_O_3_ to the Al matrix, there was an improvement in hardness, ultimate tensile strength, and impact strength [25,26,27]. Furthermore, Fazlur et al. [28] reported that the application of thermal barrier coating of Alumina-Titania, Super-Z alloy, PSZ, Zirconia Toughened Alumina (ZTA), and Alumina via a plasma spraying technique significantly improves thermal and fatigue resistance of AMCs.

Al7075/TiC is fabricated using a liquid metallurgy process. The findings revealed an enhancement in the strength and wear resistance, thereby indicating their suitability in aerospace applications [29]. To retain mechanical strength and withstand vibrations, Yan and coworkers conveyed that a natural frequency is essential for aerospace electronic components [30]. In comparison with existing alloys, the SiC/Al composites have a higher natural frequency leading to a higher lifetime of the component. The A356 composites reinforced with Al_2_O_3_, SiC and Gr exhibited a 35% increase in tensile strength and a 40% increase thereby making them a tradeoff for high-strength aircraft structures [31].

All these examples proved that the properties of Al can be altered by using several technologies along with the appropriate reinforcements in volume fractions and these can substitute the heavier existing materials in application. In recent years, significant application of AMCs has been reported in various functional and structural aircraft applications. Due to the increased prominence of fuel consumption and environmental concerns, AMCs are presently more desirable in the transportation sector.

### 2.2. Magnesium (Mg)-Based MMCs

The aggressive demand for light high-performance materials is possibly increasing with the usage of Mg-based metal matrix composites because of their lower densities. The Mg-based alloys MMCs, especially Mg-Al systems, are excellent materials for engineering lightweight structures for military and civic aircraft applications. The Mg–matrix composites can be used in aircraft, piston ring grooves, disk rotors, gearbox bearings, gears, shift forks, and connecting rods. However, their production cost is higher due to their complex manufacturing techniques. To cope with this, the usage of inexpensive reinforcement materials can provide room to maneuver this low-density material into the market. Due to their light weight, MMCs are observed as desirable materials for aircraft structures wherein weight reduction is the principal factor to be considered. However, for efficient use in the aerospace industry, further investigations are required to increase the mechanical performance of magnesium and its alloys. To produce complex structures with enhanced mechanical performance, microstructure refining in Mg-based alloys such as magnesium-lithium (Mg–Li), magnesium–zinc–zirconium (Mg–Zn–Zr), and magnesium–aluminum-zinc (Mg-Al–Zn) is carried out leading to higher plasticity. Several aircraft structures are manufactured using Mg alloys through casting and machining [32]. For the operating temperature of 250 °C, Mg alloys such as WE43B, ZE41A, EV31A, and QE22A reinforced with rare earth materials are proposed for aircraft applications [33]. Recently, jet engine manufacturers have utilized significant volumes of Mg-based alloys in aircraft structures for both military and commercial applications [14]. Magnesium alloys manufactured using the process of investment casting provide enhanced mechanical performance [34]. Furthermore, the reinforcements such as B_4_C, Al_2_O_3_, and SiC are added to the matrix to improve the tribological and mechanical properties of magnesium alloys [35]. In addition, by the process of electroplating, the hardness of chromium-coated magnesium alloy AZ31 is increased from 49 to 53 BHN [36]. Another approach to improving their performance at elevated temperatures is the incorporation of thermally stable reinforcements. AM60 and AZ91 are presently the most widely investigated Mg-Al alloys matrix for Mg matrix composites, due to their prevalent usage in the automotive industry. The reinforcements such as ceramics particles due to their higher strengths, hardness, elastic modulus, thermal stability, and lower densities are mostly preferred for Mg–matrix composites. Magnesium matrix composites reinforced titanium diboride particles resulting in an increase in the hardness and compression strength of composites mainly due to the inclusion of hard ceramic particles and it can be considered as most suitable for aerospace engineering [37]. Furthermore, Muhammad et al. [38] analyzed the impact of alumina and SiC reinforcements on the mechanical properties of Mg alloy. The hardness improved with the increase in the percentage of reinforcement. However, these reinforcements exhibited some limitations such as lower compatibility, low ductility, and wettability with the Mg matrix.

### 2.3. Titanium (Ti)-Based MMCs

Titanium matrix composites (TMCs) consist of Ti alloys as the matrix material. Due to their excellent corrosion resistance and high strength at elevated temperatures. TMCs are widely used in the aerospace, marine, and automotive industries. Titanium alloys retain their strength at even elevated temperatures as compared to Al, which is beneficial for the manufacture of aircraft and missile structures, with higher operating temperatures and speeds. TMCs reinforced with fibers are mostly used in developing aircraft structures. TMCs that have demonstrated properties suitable for aerospace applications mostly consist of the conventional (Ti6A12Sn4Zr2Mo, Ti6A14V, and so on.) and advanced (TiAl, Ti_3_A1, and so on) Ti matrix alloys that are reinforced with continuous arrays of 30–40% vol. of SiC. These fibers possess high modulus and strength [16].

TMCs are mainly categorized into two groups based on the type of reinforcements: Continuous and discontinuous reinforced TMCs. Continuously reinforced TMCs were produced by the inclusion of SiC-coated boron fibers as reinforcements called Borosicfibers [39]. As these fibers were costly, their usage as reinforcements was discontinued and replaced by carbon fibers and silicon fibers [40]. The behavior of these fiber-reinforced composites has not yet been studied extensively for high-performance applications [41]. Discontinuously reinforced TMCs exhibited higher specific stiffness, specific strength, thermal stability, wear resistance, and high-temperature stability as compared to the conventional Ti-alloys. These superior properties increase their applicability in the aerospace industry. Several particulates are preferred as reinforcements for TMCs that include B_4_C, TiB_2_ ZrC, TiB, TiN, Al_2_O_3,_ SiC, and TiC. Among these TiC and TiB_2_ are mostly used [42] and other reinforcements including nano SiC [43], Si_3_N_4_ [44], nano Al_2_O_3_ [45], and carbon nanotubes [46] are also found in the literature. As compared to continuous fiber-reinforced TMCs, the production cost of DRTCs is low. Huang et al. [47] revealed that one of the efficient ways to enhance the ductility, deformability, and high-temperature strength of PM-fabricated DRTCs is by tailoring the reinforcement distribution. This led to an improvement in the ductile nature and enhanced the tensile strength at room and high temperatures. Moreover, Cui et al. [48] fabricated TiAl alloy composites reinforced with carbon fibers coated with graphene by powder metallurgy, melt spun, and vacuum melting techniques. The fabricated composites exhibited good fracture strain, excellent strength, and microhardness thereby predicting this approach as simpler and more advantageous to fabricate fiber-reinforced TMCs. Liu et al. [49] effectively designed and developed an in situ Ti64 matrix composite reinforced with TiC particle, ultrafine Ti_5_Si_3_ needle, and Ti_3_SiC_2_ bar. The result showed the developed composites exhibited good ductility and strength as compared to the monolithic Ti64 alloy. The properties improved mainly as a result of the substantial size of the matrix region, hybrid and solid solution strengthening effect, and tailored network structure.

Furthermore, Kim et al. [50] developed B_4_C-reinforced titanium matrix (TiB + TiC) composites via vacuum induction melting and achieved better friction and wear behavior at 20% of reinforcement content. Additionally, An et al. [51] successfully developed in situ Ti64 composites reinforced with TiB by powder metallurgical process. The hardness and wear properties are remarkably enhanced as a result of TiB addition forming a network boundary that acts as a “barrier wall” and effectively resisted abrasion as compared to the Ti64 alloy. In another study, Chaudhari et al. [52] fabricated TiB and TiC-reinforced Ti-4Al-2Fe by spark plasma sintering (SPS). There was a unique distribution of reinforcements with fine needles of TiB near the surface, ultrafine TiC on top, and coarser TiB whisker in the bulk. The TiC layer on the surface exhibited the maximum hardness. Thus, these examples show that the specific wear characteristics of the DRTCs can be improved by systematic control of the microstructure and volume fraction of reinforcement. Extensive research work has been carried out on the toughening mechanisms of TMCs reinforced with fibers [53,54]. Yanqing et al. [55] studied the effect of the addition of SiC fibers (uniaxially) on the fracture toughness of the Ti64 alloy. The study revealed that the fracture toughness decreased upon heat treatment as a result of an interfacial reaction between the Ti64 matrix and SiC fibers [55]. The size (diameter) of the fiber was found to affect the fracture toughness of metal composites [56].

**Table 1:** Properties and applications of metal matrix composites in aircraft.

Matrix Material	Reinforcement Material	Properties	Application	References
Titanium	SiC	-High-impact energy -Weight reduction (32%)	Landing gear	[57]
Al	Cu–Nb,	-Improved high-temperature strength	Engines	[58]
Al alloy (LM25)	SiC	-Light-weight -Optimum performance -Reduces fuel costs	Aircraft wing	[59]
Al alloy	SiC	-Low density -High elastic modulus -High thermal conductivity -Preventability of resonance vibration	Fuel tank (door part) and fans (F-16 fighter aircraft)	[30]
Al alloy (AA6061)	Activated carbon	-Good thermal resistance	Engines	[60]
Cu	Nb3Sn	-Creep resistance -Stiffness	Engines	[61]

### 2.4. Manufacturing of MMCs

The MMCs’ manufacturing techniques are simply established on the state of the matrix in the processing technique such as liquid state processing, solid state processing, and gaseous state processing.

#### 2.4.1. Liquid State Processing

In alloys with a low melting point such as AL/Mg, the liquid state processing technique is highly convenient because it can produce a shape close to the mesh at a lower production cost. The particles or (short) reinforcing fibers can be mixed with the molten matrix before casting, to acquire a composite structure. The process of stirring is usually required to substantiate that the subsequent material is less uneven. Traditional foundries are employed to form composite ingots, which can be processed into extruded billets or rolled billets for further processing. Continuous casting produces long semi-finished products with constant sections or bars. The heterogeneity obtained as a result of these technologies is a common problem, however, this can be solved by deformation processing or grouping of regions with low concentrations.

**Figure 1:**
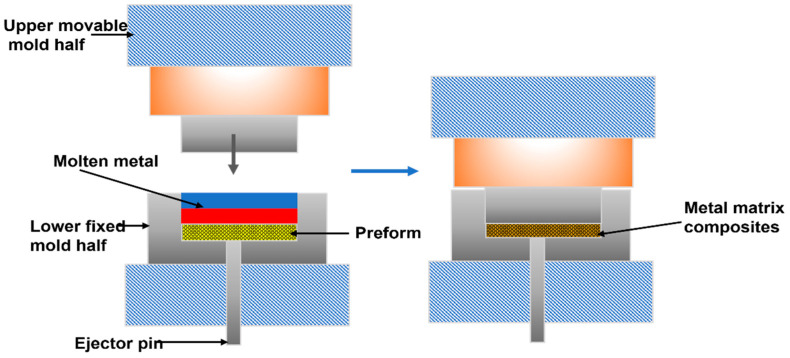
Schematic illustration of squeeze casting technique.

Sun et al. [62] used ultrasonic cavitation to disperse and treat carbon-coated Ni nanoparticles in molten magnesium to manufacture finely reinforced composites with up to 4.9% by weight Ni. Another method utilizes liquid metal to infiltrate and reinforce the preform such as non-pressure infiltration, compression molding, and low-pressure infiltration. The non-pressure infiltration utilizes 55–57% of SiC particle preforms with Al alloy ingots placed on them heats them to 790–810 °C depending on the thickness of the powder bed of SiC and exposes them to a nitrogen atmosphere for 212 h [30]. This is a cost-effective technique for fabricating lightweight and high-compression strength composites for aerospace applications [63]. Squeeze casting as illustrated in Figure 1, is a method of pressure-assisted infiltration of particles or short fibers performed through liquid metal. Compared to traditional infiltration, it has a shorter processing time, produces a relatively complex shape, and minimal porosity improves wettability, good dimensional precision, and minimization interfacial reactions [64,65]. Carbon fiber-reinforced Al composites with better toughness, hardness, strength and better wettability have been successfully achieved by using this technique [66].

#### 2.4.2. Solid-State Processing

The solid-state-based processes mainly involve powder metallurgy (PM). The reinforcing material in the form of fine powder is intimately mixed with the metal alloy, then cold-pressed followed by hot-pressing or sintering. Due to the grain and fiber arrangement, secondary processing (such as forging or extrusion) is usually used to achieve a fully compact composite material with improved properties. PM can be used to produce discontinuous fiber-reinforced composite materials that eventually contain nanoparticles. The benefit of this method is that it can produce parts with a shape close to the web. Furthermore, functionally graded materials can be obtained by gradually increasing or decreasing the reinforcement volume in a specific area of the component to be developed. One disadvantage is that it is difficult to control the spread of reinforcing steel, resulting in fewer reinforcing steel clusters and areas.

The vacuum hot pressing (MCF method) was employed for producing SiC-reinforced TiMC. Silicon carbide monofilaments, 140 mm in diameter, are coated with Ti6Al4V with a thickness of 50 mm, and then stacked together in hexagonal and square arrays. The fiber distribution in MCF was very uniform, and the fiber volume fraction can reach up to 80%. The research on the MCF method mainly focuses on the consolidation behavior of MCF, to optimize processing parameters. However, the major problem lies in the processing of highly active titanium alloys with reinforcements.

#### 2.4.3. Vapor Deposition

**Figure 2:**
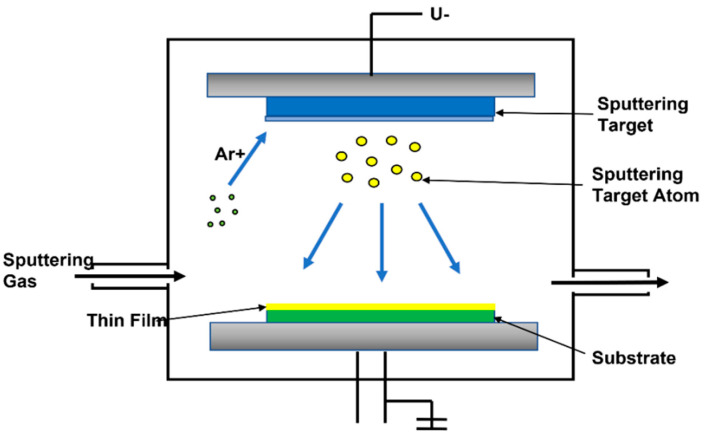
Schematic illustration of physical vapor deposition technique.

The gaseous treatment is carried out mainly utilizing plasma spraying, such as metal-coated fibers. The process is characterized by the matrix deposition on the individual fibers of the vapor phase. The manufacture of composite materials is carried out utilizing hot isostatic pressing operations. The PVD coating on the mechanical components of the jet engine prevents wear. PVD coating has high hardness and low friction, making it an ideal functional metal coating in the aerospace industry. Fluctuating temperatures from negative temperatures to hundreds of degrees Celsius require metal coatings that can withstand extreme conditions. PVD was chosen because of its thermal stability and corrosion resistance, making it an excellent choice for finishing aerospace metals. Thermal barrier coatings for aircraft engines have been developed by the PVD technique as shown in Figure 2.

### 2.5. Applications of MMCs in Aircraft Components

The comprehensive attributes and manufacturing costs of MMC vary greatly based on material properties, processing methods, and product quality. In engineering, the types of composite materials used, and their applications vary widely, as do the properties that decide their selection in applications. For example, in the aerospace industry, the properties such as low cost, high weldability, and high specific modulus of extruded alumina-reinforced Al are required. MMCs are used in various applications, including aerospace due to their unique characteristics as demonstrated in Table 1. Applications include engine components, brake components, and drive shafts. The transport sector being a cost-sensitive sector is their major limitation. Therefore, by reducing the manufacturing cost of MMC, the traditional components can be replaced by MMC components. The application of MMC in the aerospace industry is due to their ability to provide enhanced specific strength and stiffness which considerably improve aircraft performance. MMCs are used primarily in military and commercial aircraft. For example, on the F16 aircraft, the aluminum access doors have been substituted by MMC reinforced with SiC particles, thus improving fatigue life. Due to its high fatigue resistance, specific stiffness, and strength, continuous fiber-reinforced MMC has also been used in military applications. Titanium-based composites reinforced with SiC monofilament have been used as the F119 engine nozzle actuator control device in the F16 [67]. MMC replaced the heavier Inconel 718 used in the actuator rod and the stainless steel in the piston rod [68]. MMC replaces carbon/epoxy composites that have foreign body damage (FOD) problems. The Boeing 787 was the first commercial jet aircraft made primarily of composite materials [69].

**Figure 3:**
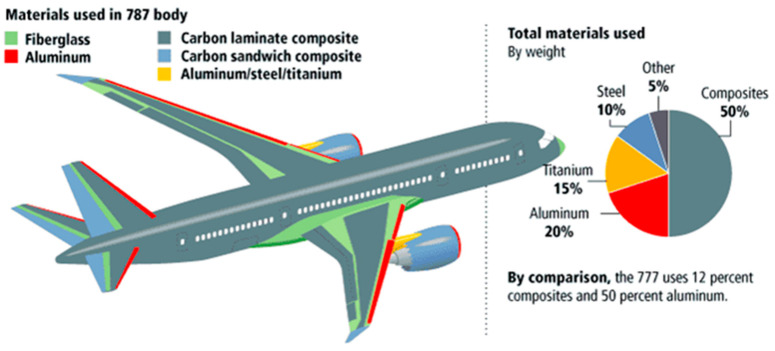
Overall distribution of composite materials used in Boing 787 aircraft (Reprinted/adapted with permission from Ref. [70]).

The Boeing 787 uses more composite materials in the main structure and fuselage than any prior Boeing commercial aircraft, as shown in Figure 3 [70]. The Boeing 787 is comprised of 80% composite material by volume. The material composition is 50% composite, 20% aluminum, 15% titanium, 10% steel, and 5% other by weight. Performing the design process without preconceptions allowed Boeing engineers to identify the best materials for the specific application of the entire airframe. As a result, almost half of the fuselage is composed of carbon fiber-reinforced plastic and other composite materials. Compared with more traditional Al designs, this method can reduce the weight by an average of 20% [71]. Still, there are many reasons to consider the usage of lightweight Al compounds.

## 3. Ceramics Matrix Composites

Ceramic matrix composites (CMC) have been proposed for aircraft structures that require high strength and fracture toughness. In addition, they are characterized by lightweight, low thermal expansion, high temperature, and oxidation resistance, and resistance to catastrophic failure. Compared with traditional engineering materials such as metals, CMCs are much more resistant to aggressive environments and high temperatures. In CMCs, the ceramic forms the matrix material, generally a technical ceramic, which is manufactured by a relatively complex process from raw materials with small particle size (micron or nanometer), high purity, and good mechanical, thermal, and electrical resistance. Ceramics usually form mixed chemical bonds between ionic and covalent. They have high hardness, chemical stability, low density, and fire resistance (that is, they maintain mechanical strength at high temperatures). Table 2 shows the properties and compositions of various CMCs used in aircraft.

In CMC, the “reinforcing phase” can be fibers, whiskers, and continuous particles. The characteristics of the resultant CMCs are determined by the volume fraction, distribution frequency, size, orientation, and geometry of the reinforcement phase. Current CMC applications include aerospace structures, high-temperature trim, faceplates, internal combustion engines, and turbines as mentioned in Table 2. CMC is now being introduced into many new areas, the production cost is significantly reduced, and its application range will be expanded. There is a great need to develop cost-effective SiC fibers to promote CMC applications where cost plays a significant role. The aircraft brakes have transitioned from organic materials (such as non-asbestos organic brake materials, and asbestos fiber-reinforced resin-based composites) to powder metallurgy materials (such as iron and copper-based metals) and carbon/composite materials carbon (carbon brakes) as demonstrated in Table 3.

**Figure 4:**
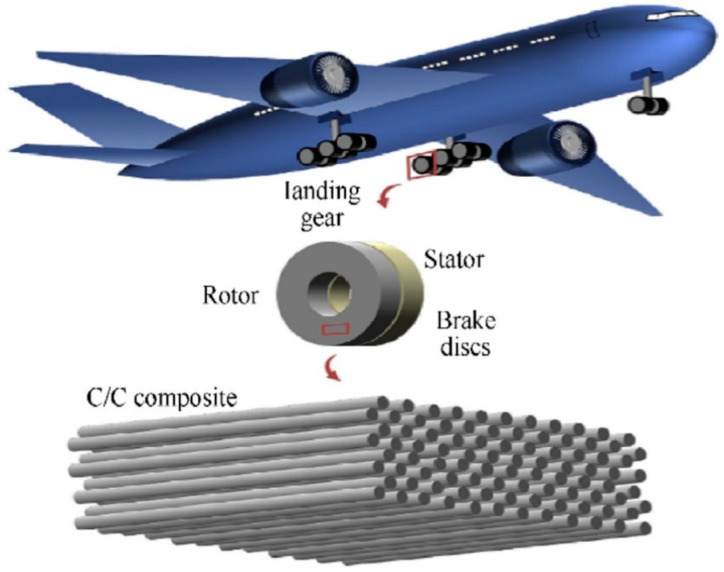
Schematic illustration of brake discs in the aircraft landing gears made with C/C material (Reprinted/adapted with permission from Ref. [92]. 2008, Elsevier).

Figure 4 shows carbon-carbon composites (unidirectionally reinforced) with different fiber orientations for aerospace applications [92]. As shown in Table 3, they have excellent high-temperature, mechanical, and thermal performance, so carbon brakes can cope with the low-temperature performance of traditional brakes. Furthermore, compared to steel brakes, carbon brakes significantly reduce the weight of the brake system, which contributes directly to reducing fuel consumption related to engine emissions. The brake system on the Boeing 737 NG is made of carbon and is 300 kg lighter than the steel brakes [93]. C/SiC composite brakes overcome these shortcomings while retaining the advantages of carbon brakes, as shown in Table 3. These brakes possess remarkable properties such as long life and low sensitivity to friction, high friction coefficient and stability, and low oxidation [94]. C/SiC brake materials have become the focus of attention as the fourth generation of aircraft brake materials. C/SiC brakes exhibit some excellent friction properties, such as a high static friction coefficient, lower sensitivity to wet conditions, low wear rate, and higher braking efficiency.

As the thrust-to-weight ratio of an aircraft engine increases, the heat flow and impact load on the high-temperature components such as nozzles, combustion chambers, and turbine components become more severe. For example, when the thrust-to-weight ratio is 10, the turbine inlet temperature reaches 1500 °C and the turbine inlet temperature can rise to 1800 °C if the thrust-to-weight ratio further increases [95]. Continuous fiber-reinforced ceramic matrix composites (CFRC CMC), such as silicon carbide fiber-reinforced ceramic matrix composites (SiC/SiC CMC) and carbon fiber reinforced ceramic matrix composites (C/SiC CMC), have low densities ranging from 2–3 g/cm^3^, high-temperature resistance up to 1600 °C, and, as compared to monolithic ceramics, higher fracture toughness [96]. Therefore, CFRC CMC is considered a promising material that meets the requirements of aero-engine hot section components. It can increase the operating temperature to 200–350 °C, thereby reducing or even replacing the cooling structure. Additionally, it effectively improves the reliability of aero-engines [97,98]. CFRC CMC has been used in the nozzles, combustion chambers, turbine stators, and other hot sections of aero-engines such as M882, F100PW229, CFM565B, F135, GEnx, LEAPX. CFRC. CMC manufacturing technology is considered to be the leading improvement of aero-engine hot-segment components, so it is highly valued by developed countries and regions such as Europe, America and Russia [99].

### 3.1. Manufacturing of CMCs

Several methods can be used to process CMC via liquid, solid, or gaseous precursors. Depending on the difference in the coefficient of thermal expansion, tensile stress is generated in the matrix around the reinforcing material that suppresses densification. Due to this, the quantity of reinforcement is generally kept below 40% by volume [100].

#### 3.1.1. Reaction Sintering Process

The reaction sintering process is employed for the production of CMC [101]. In this process, the ceramic particles for instance when carbon fibers are added to Si, infiltrates at low pressure and at the temperature of 1700 °C to produce liquid silicon resulting in a reaction between Si and carbon to form a thin matrix, resulting into excellent thermochemical compatibility between reinforcement and matrix [101,102]. Using this technology, TiB_2_SiC ceramics reinforced with multi-walled carbon nanotubes (MWCNT) were developed and the thermal shock resistance of the material improved significantly [103].

#### 3.1.2. Liquid Infiltration Method

**Figure 5:**
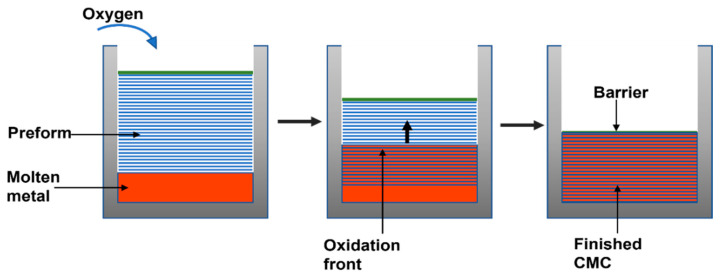
Schematic illustration of the direct oxidation process.

The liquid infiltration method is similar to the technology used for metal or polymer infiltration. The pre-formed reinforcement phase is penetrated by the liquid or matrix precursor suspension under vacuum or external pressure by capillary action. For example, glass matrix composites are developed by this technique, which contains various fibers (SiC, C, Al_2_O_3,_ and mullite) that are extracted with molten glass in a crucible. Other materials are those produced by the infiltration of hydrocarbons (pitch or phenolic resin) or organometallic polymers, which are subsequently pyrolyzed to produce SiC and a carbon matrix, respectively. Figure 5 shows the process of direct oxidation required for complete densification.

#### 3.1.3. Sol-Gel Process

The sol-gel process involves a low processing temperature and high compositional uniformity. Initially, when nano-sized particles with a radius of up to 100 nm (such as ceramic particles) are precipitated in a liquid (water or organic solvent), the colloidal suspension is formed because of the chemical reaction. These liquid sols easily penetrate the perform due to their low viscosity. Polymerization turns the sol into a gel. The gel can become ceramic at a relatively low temperature, thereby reducing the possibility of damage to the reinforcing fibers. Since the ceramic content in the gel is relatively low, it will shrink significantly after drying. The densification of the ceramic matrix is usually increased by repeated infiltration and drying cycles until the desired density is reached. The volumetric performance of sol-gel ceramics can be further improved by adding ceramic particles. These particles also reduce the formation of cracks during the drying phase. However, the difference in shrinkage between the steel bar and the base is large, which can lead to cracks. Finally, the high-temperature self-propagating synthesis (SHS) technology, which is mainly used to make porous refractories, can be used to produce CMC as SiC (whisker) and Al_2_O_3_. The pressure is applied shortly after or during the exothermic reaction inside the matrix to densify the material [104].

#### 3.1.4. Chemical Vapor Infiltration (CVI)

**Figure 6:**
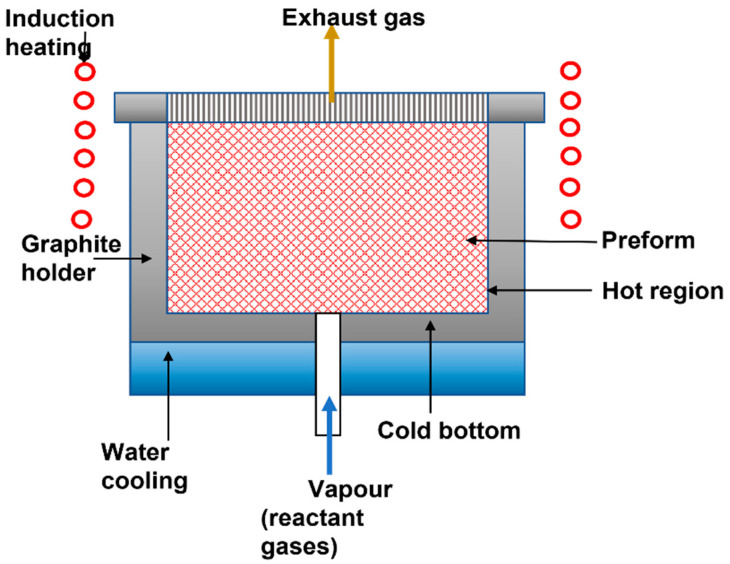
Schematic illustration of chemical vapor infiltration process.

Chemical Vapor Infiltration (CVI), as illustrated in Figure 6, is a process involving lower processing temperatures than liquid infiltration, thereby avoiding fiber degradation. However, the airflow in the preform can cause pore occlusion, requiring multiple impregnation and processing cycles to completely close the pores. CVI was originally used to make carbon/carbon composites through the pyrolysis of CH4 at 1000–2000 °C. CVI of fiber preforms is a method used in CMC, the matrix of which is SiC, Si_3_N_4_, C, B_4_C, TiC, and Al_2_O_3_, as well as Nicalon (SiC) and Nextel (a type of Al_2_O_3_) reinforcement materials. CVI is an extension of CVD technology. When CVD is employed to incorporate a considerable amount of matrix material into the fiber preform, it is called chemical vapor infiltration or infiltration. The CVI process has been modified as microwave-enhanced CVI (MECVI) and two alternative pre-filtration steps, vacuum bagging, and electrophoretic infiltration, to reduce the time of the CVI process and reduce the cost of this usually expensive process. The system studied is based on silicon carbide fibers in a silicon carbide matrix (SiC/SiC). The vacuum bagging (VB) allows a better way of bonding the matrix particles to the intertow region. When SiC of larger particle size is used, there is a reduction in infiltration of the intratow region.

### 3.2. Applications of CMCs in Aircraft Components

Although many monolithic ceramic materials exhibit inherent properties, the main problems associated with their use in aircraft engines are their sensitivity to defects and their brittle fracture mode. Continuous fiber CMCs are a class of interesting material because (i) they have high-temperature performance compared to superalloys; (ii) as compared with monolithic ceramics, CMCs possess a higher fracture toughness and can be used where structural integrity is more necessary. Therefore, CMCs have great potential to meet the general requirements of these aircraft engines. They can achieve higher material temperatures, the introduction of thermal barrier coating (TBC), and air-cooled sheets thereby discarding the usage of cooling air to improve the performance. Of course, to successfully implement CMCs in aero-engines, the overall benefits of the system must be considered. In addition, CMCs can significantly reduce weight, thus their T potential applications include non-structural and structural components of aircraft engine components (refer to engine layout) [105]. Table 2 shows the various ceramic matrix composites used in various aircraft applications.

#### 3.2.1. Turbine Blades

**Figure 7:**
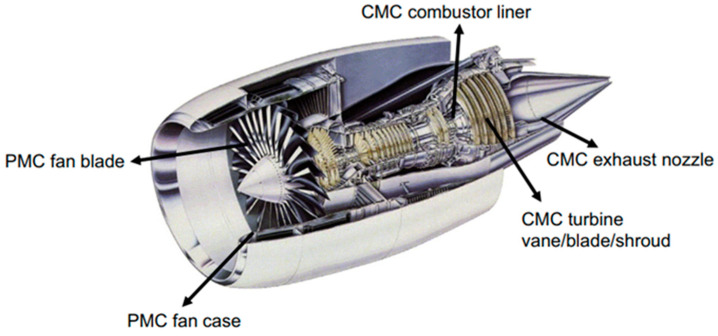
Cross section of a gas turbine showing components made of CMCs (Reprinted/adapted with permission from Ref. [108]. 2016, Elsevier).

The materials withstanding elevated temperature are especially required for gas turbine blades as shown in Figure 7. Carbon/carbon (CC) composite turbine blades retain the strength at about 1050 °C of the turbine exhaust gases and are very light in nature. These characteristics make the aircraft possible to achieve speeds of Mach 10 [106]. In contrast, titanium-based composites can only reach Mach 3.8 (working temperature 450 °C). Taking into account the specific tensile strength (s/r) of CC compounds made of alternating layers of carbon blankets and unidirectional fibers can reach 160 MPa/g cm^3^ at 2000 °C, while the specific strength tensile strength of traditional ceramics reaches 40 MPa/g cm^3^ up to 1200 °C [107]. SiC/Al_2_O_3_ or SiC (fiber)/Si_3_N_4_ CMC as a substitute for C/C composite materials exhibits poor performance (60 MPa/g cm^3^). In addition, the SiC-coated carbon fiber composites in a carbon matrix are the high-performance materials that are preferred in the aerospace industry as mentioned in Table 2. The high-performance oxide composite (HIPOC) was launched in 2009 and focused on the development of several oxide-based CMCs for hot segment applications in aircraft turbines or ground engines [107].

#### 3.2.2. Braking System

The braking system is currently an important field in the automotive and aviation industries. On activation, the brake responds to hydraulic pressure through the disc (rotor and stator) and the friction generated causes the surface temperature of the component volume to reach 3000 °C and 1500 °C. C/C composites as compared to traditional systems (high-strength steel and sintered metal) result in significant weight reduction. By applying this material to the braking system of commercial aircraft, the economic weight can be reduced from 1100 to 700 kg. Therefore, it not only improves the properties of the materials, such as resistance or environmental stability, but also the reproducibility and reliability of the process, as well as the reduction in fabrication costs [107].

#### 3.2.3. Blisks (Blade Discs)

The design of blisks (rotating parts) is strongly driven by a force/density ratio that is different from that of static components. Lightweight blisks eliminate extra weight, reducing axle loads, bearing chamber loads, etc. These series of effects can bring much greater system benefits than the CMC application alone. The maximum tensile strength at room temperature is almost 500 MPa. Three-dimensional woven fabric discs using continuous Tyrannoe Si-Ti-C-O (LOXM grade) are densified by using combined techniques of chemical vapor infiltration (CVI) and polymer impregnation and pyrolysis (PIP).

#### 3.2.4. Exhaust Nozzle

Several companies are evaluating the use of CMC Ox-oxide (Ox/Ox)-based exhaust nozzles to improve component durability in subsonic jet engines (compared to titanium) and avoid the weight increase associated with the use of higher metal alloys. Boeing is developing Nextel 610/aluminosilicate composite acoustic cores and exhaust nozzles for commercial aircraft. GE Aviation has invested heavily in Ox/Ox compounds, and Ox/Ox material was initially used as the divergent exhaust seal of the F414 engine [109]. A rust CMC exhaust ground test demonstrator was used for future large-scale civil transportation (High-Speed Civil Transportation (HSCT)), supersonic aircraft [110]. 

#### 3.2.5. Turbine Nozzle Blades

Turbine nozzle blades have complex shapes. A slip/hip mold casting of SiC whiskers and silicon nitride (Si_3_N_4_) powder was used for shaping research as shown in Figure 7 [111]. However, a few essential technologies such as the development of material systems (thermal stability of non-oxidized silicon carbide fibers, matrix, and interface), the establishment of design methods, low-cost manufacturing processes, and the development of non-destructive evaluation techniques need to be further developed before they can be used widely in CMC. GE aviation tested the world’s first rotating SiC matrix CMC material for low-pressure turbine blades of F414 engines [112]. In an approach to double the use of CMC engine parts in aircraft, projects have been initiated where materials that can withstand higher temperatures and are weight-saving requiring no need for cooling air would be preferred [113].

## 4. Polymer Matrix Composites

**Figure 8:**
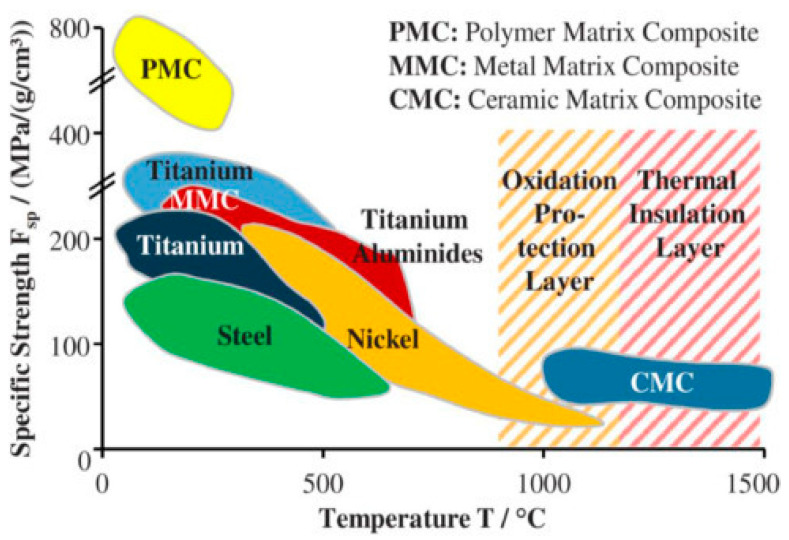
Specific strength of aircraft engine materials as a function of temperature [118]. (Reprinted/adapted with permission from Ref. [118]. 2013, Elsevier).

Polymer-matrix composites (PMCs) are one of the lightest composite materials. These materials have been used on a large scale in current developments for military combat aircraft, small and large civil transport aircraft, and helicopters [114]. The extensive use of these compounds in the current developments of the stated machinery is a brilliant example of using the potential of such composite materials [115,116,117]. As evident from Figure 8, polymer matrix composites exhibit high strength, however, they can be used only at low operating temperatures [118].

Even in a conservative design, as shown in Table 4, it is easy to achieve a 30% weight reduction. However, recently, the use of reinforced plastics in aircraft components was limited to secondary frame fuselage components made of fiberglass/epoxy or fiberglass/polyester. Currently, with the development of advanced aramid and graphite fibers, the application of advanced fiber/epoxy resin composites is mainly focused on the main fuselage structure.

A study was conducted to manufacture samples of LSU03 aircraft propeller products using fiber/epoxy resin through two manufacturing methods, namely hand lamination and vacuum-assisted resin transfer molding (VARTM). The epoxy matrix supports the fibers and binds them together in the composite. The matrix transfers any load applied to the fibers, maintains the fibers in their selected position and direction, gives the composite material environmental resistance, and determines the maximum temperature of use of the composite material [119]. Relatively low glass transition temperature and limited thermal oxidation stability limit the use of fiber/epoxy composites. Compared to fiber-reinforced epoxy resins, also known as high-temperature resistant polymers, they provide the opportunity to increase the temperature of use by almost double, but their properties are difficult to handle. Early high-temperature resin technology increased the possibility of manufacturing structural parts. The existence of these voids or defects severely reduced the mechanical properties and the stability of the thermal oxidation of composite materials [120,121].

In the progress of research on polymers to develop high-performance resin as a matrix material to meet the challenges of designing the complex design parts of modern aircraft, carbon fiber has been preferred as a strong reinforced material. Carbon fiber-reinforced polymer (CFRP) composites have been extensively used in aircraft structures due to their light weight, high durability, good thermal resistance, and good mechanical, tribological, and electrical properties [122,123]. Some researchers developed nano-structured composites with superior dielectric and mechanical properties for aircraft applications by reinforcing different types of carbon nanotube (single, double, and multiwalled) in the epoxy matrix [124]. Other fibers, such as graphite fibers, kenaf fibers, glass fibers, ramie fibers, etc., were also added to the polymer matrixes to develop composites for aircraft applications as mentioned in Table 4.

### 4.1. Manufacturing of PMCs

PMC is very popular due to its low cost and simple manufacturing method. Several variables are considered when designing a PMC. These include not only the types of molds and steel bars, but also their relative proportions, the geometry of the steel bars, and the nature of the interface. The variables must be carefully controlled to develop structural materials optimized for their conditions of use. Common processing techniques for polymer-based compounds are as follows:

#### 4.1.1. Injection Molding

The injection molding technique is used for the fabrication of polymers and plastics [125,126,127,128]. This technique has various types including water-assisted molding, gas-assisted molding, injection foam molding, compression injection molding, micro-injection molding, and low-pressure molding [129,130,131,132]. Injection molding can produce high-precision composite parts with the least cycle time. Generally, the injection molding process involves fiber composite material in the form of particles that are fed using a hopper and then transported by a screw with a heated barrel. Once the material in the barrel reaches the required amount, the screw injects the material via a nozzle into the mold followed by cooling it to obtain the desired shape [133,134,135,136]. The final product obtained is mold-shaped and of the same size as that of the mold. These products are often accompanied by defects, such as sprays, short shots, sags, flow marks, floating fibers, and weld marks. The defects can be treated by spray-coating; however, this increases the manufacturing costs and time [137]. It is the widely used method to manufacture polymer composites reinforced with carbon fibers [138,139].

#### 4.1.2. Resin Transfer Molding (RTM)

**Figure 9:**
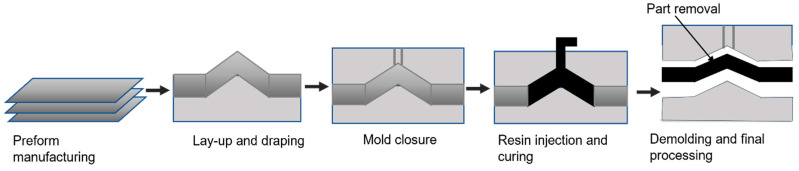
Resin transfer molding process for fabrication of PMCs.

RTM can manufacture large and complex 3D parts with improved mechanical properties, high surface finish, and small dimensional tolerances. RTM is a rigid closed mold process. In this technique, the lamination sequence is positioned in a cavity, the thickness of the part is determined between two closed mold halves and the resin is injected under pressure. Once the resin reaches the vent, the gate is fastened followed by the impregnation of the preform. After curing, the mold is opened and closed, and the part is taken out. These steps are outlined as evident in Figure 9. Vacuum-assisted resin transfer (VARTM) molding is the advanced form of RTM in which preformed fibers are positioned in a mold followed by a perforated tube placed between the vacuum bag and the resin container. The vacuum force draws the resin in the fiber through the perforated tube to combine with the laminated structure [140]. Thermosetting resins are mostly the preferred matrix used in RTM due to their low viscosity during processing. Among thermosetting resins, there are several types of aerospace applications suitable for RTM. For example, epoxy resins, phenolic resins, cyanate esters, and bismaleimide epoxy resins are commonly used in the development of aerospace composites, especially carbon fiber-reinforced epoxy resin laminate [141]. The wide variety of epoxy resins and curing agents enhance the versatility of these systems regarding the manufacturing process and the physical properties that can be obtained [142]. The heavy-loaded primary aircraft structures are fabricated using this technique ensuring high-quality and low-cost production [143].

#### 4.1.3. Compression Molding

Compression molding consists of preheated molds that are mounted on mechanical or hydraulic presses. The backing made of prepreg is positioned between the two mold halves, and then they are pushed against each other to obtain the desired mold shape. It has a high degree of productivity, short cycle time, and dimensional stability, so it has been used in various applications in the automotive industry [144,145]. Carbon fiber-reinforced PEEK polymer is the main structure, and element, used in, for example, supports, hinges or accessories developed for aerospace applications using compression molding technology [146].

#### 4.1.4. Laying Prepreg

Laying prepreg is the blend of fiber and uncured resin, prepreg with thermoplastic or thermosetting resin material, requiring temperature activation. These prepregs are ready-to-use materials in which the easily impregnated layer is cut and placed in the open mold [147]. VORAFUSE is a technology developed by Dow Automotive Systems that combines carbon fiber and epoxy resin for prepreg applications to improve cycle time and material handling in the compression molding of composite structures. They cooperated with several automobile companies to significantly reduce the weight and thus efficiently manufacture the CFRP composite structure [148]. Initially, the fiber preform is positioned in a mold and a thin release layer is applied to the mold to facilitate removal. A brush is used to mold or apply the resin material to the reinforcements. The rollers are used to press the resin into the fabric to ensure the interaction between the continuous reinforced layer and the matrix material [149,150].

#### 4.1.5. Pultrusion

**Figure 10:**
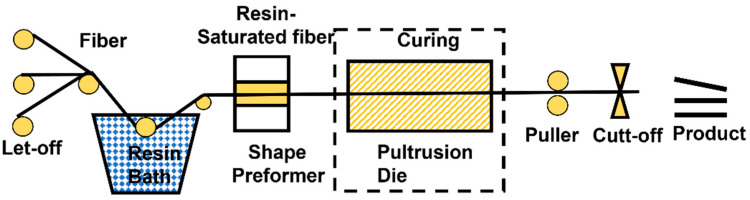
Pultrusion process for the fabrication of polymer matrix composites (adapted and modified from [151]).

The pultrusion process is the continuous passage of resin-impregnated fibers or other preforms through a mold at a certain speed to gradually mold and cure the composite part as shown in Figure 10 [151]. The pultrusion process is a low-cost method and suitable for fast-curing resins that can be used to produce parts with constant cross-sections [152]. This is a continuous process that can be used to manufacture composite materials with constant cross-sections and relatively long lengths, thus allowing for a lower cost of production and a high degree of automation [147].

### 4.2. Applications of PMCs in Aircraft Components

Various polymers including PLA, PP, and epoxy resins were employed as matrix materials for the development of composites for aircraft applications as mentioned in Table 4. Fiberglass/epoxy materials were employed to produce non-crucial components such as shroud panels, fans, duct fairings, spacers, and seals. However, CFRP finds the most practical application in aircraft component design. CFRP is employed in various aircraft due to their light weight and ability to withstand the desired conditions as evident from Table 4. A 4-seater aircraft helped to reduce the weight by almost 25% as compared to a metal alloy’s counterpart by using PMCs [153]. Moreover, in an approach to reduce the weight of the aircraft, the components were integrated and made as one composite part, such as the landing gear integrated with the fuselage, in the main landing gear bay. This mainly comprised CFRP and limited the use of titanium [154]. The component was prepared by a one-shot curing process, and it can reduce the assembly recurrent cost by up to 80% [155,156].

**Table 4:** Polymer matrix composites and their reinforcements in various aircraft applications.

Matrix	Reinforcements	Properties	Applications	Ref
Polymer	Hybrid kenaf/glass fiber	-High specific strength -Rain erosion resistance	-Aircraft brakes	[157]
Polypropylene	Hybrid bamboo/glass fiber	-Improved tensile strength -Increased fatigue life	-Aircraft structures	[158]
Polymer	Ramie fiber	-Reduction in weight (12–14%)	-Aircraft wing boxes	[159]
Polymer	Carbon fiber	-Design flexibility -High stiffness -Reduced scrap -Resistance to flames and heat -Fatigue resistance -Corrosion resistance -High strength -Damage and impact tolerance -Vibration-damping properties -Fracture resistance	-Aircraft brakes -Fuselage -Window frames -Aircraft wing -Rotors -Brackets -Boxes -Bulkheads -Fittings -Airframe -Blades -Vertical fins -Tail assemblies -Food tray arms	[160]
Polylactic acid (PLA)	Glass fiber	-Improved flexural properties -Improved tensile properties	-Engine access door -Acoustic liners -Vanes	[161]
Epoxy resin	Fiber	-Flame retardant -Good mechanical performance -Resistance to irradiation	-Aircraft structures	[162]
Epoxy resin	Carbon black	-Improved mechanical strength -Resistant to oxidation -Flame retardant	-Controlling static electricity in the avionics system	[163]
Epoxy resin	Epoxy resin Carbon fiber/S2-Glass fiber	-High OHT (open hole tension) strength -High deformation before fracture	-Aircraft structural framework	[164]
Silicone	Nano-carbon (graphene, carbon nanotube, and carbon black)	-Excellent performance at different temperature ranges -Resistant to chemicals, and aging, -Unique electrical insulation properties -Excellent resistance to oxidation	-Aircraft structure	[165]
Polymer	Carbon fiber	-Toughness -Durability	-Lockheed Martin F-35 (lighter fighter aircraft wings, horizontal fuselage, vertical and horizontal stabilizers)	[166]
Thermoset and thermoplastic resins	Carbon fabrics, glass fabrics, and Kevlar fabrics	-Lightweight -Negative refractive index -Negative permittivity, and permeability	-Radar-absorbing structures (stealth aircraft)	[167]

### 4.3. Filler Dispersion Methods in Polymer Composite Processing

Fillers have been added to the matrix in recent years to develop a novel composite, which meets the functional requirements. Although composite processing processes vary, all must address the following challenges, which have a direct impact on the characteristics of the composites: alignment, dispersion, and functionalization. Polymer composites are often processed using the following techniques:

1. Solution processing
2. In situ polymerization
3. Melt-mixing

#### 4.3.1. Solution Processing

The fillers are initially spread in a solvent or solution of the polymer. Then an energetic agitation such as magnetic stirring [168,169], sonication or reflux [170,171], and high shear mixing [172] can be used to mix the solution.

Mechanical or high-speed stirring is the easiest and most commonly used method to scatter the fillers in the matrix. The fillers are directly mixed into the polymer matrix and the mixture is continuously stirred for a fixed time to disperse the particles in the matrix. The stirring can be achieved through a magnetic field or by using a motor.

The sonication method uses ultrasound waves to stir filler particles in the polymer matrix. It is often performed with an ultrasonic bath or a probe/horn, also known as a sonicator. The ultrasound promulgates through a series of contractions over the process of sonication. The created attenuation moves through the medium of the polymer, facilitating the dispersion of the particles. As a consequence, individual particles are separated, allowing for high-quality dispersion. Whereas this sonication technique may occasionally cause structural damage to the filler particles, there are also methods for dispersing the particles without causing harm. High-shear mixing methods comprise the usage of a three-roll mill, where the filler/polymer mixture is fed between the center and feed rollers and then collected from an apron roller. The shearing of the filler particles can occur as the material passes between the rollers.

#### 4.3.2. In Situ Polymerization

The in situ polymerization [173,174] technique generally involves dispersing the particles of filler in a neat monomer (or several monomers) or a solution of monomer, followed by polymerization of the dispersed filler. These efforts are frequently followed by extraction/precipitation or solution casting to create models for testing.

#### 4.3.3. Melt-Mixing

Melt mixing [175,176] involves the mixing of a polymer melt with a filler (dry powder) under high-shear conditions. Pellets of polymer are melted to produce a viscous liquid during the process. An extruder or a high-shear mixer is subsequently used to combine the nanoparticles into the liquid polymer. Injection molding, compression molding, or extrusion can be used to create the final bulk nano composite samples.

The conventional approaches for determining the state of nanoparticle dispersion are primarily qualitative and involve a visual examination of images from optical microscopy [177,178] transmission electron microscopy (TEM) [179], scanning electron microscopy (SEM) [170,172], or scanning probe microscopy (SPM) [180].

## 5. Properties of PMCs, MMCs, and CMCs Required for Aircraft Applications

Materials for aircraft applications must possess high strength, and be creep-resistant, fracture-tough, durable, damage-tolerant, and lightweight. The Boeing 747, for example, requires over 6,000,000 components from various material systems and suppliers around the world. Composites offer a reduction in weight, fatigue, and corrosion, lower part count, tailorable strength and stiffness. The various components of aircraft demand a different set of properties. For example, the primary drivers for the design of fuselage are damage tolerance and durability. The leading drivers are crack initiation and growth rate, fracture toughness, and fatigue, although, strength, stiffness, and corrosion are the key parameters. Similarly, the wing design demands high strength, damage tolerance and durability. Meanwhile, strength, fatigue, and damage tolerance are highly important for the propulsion structures and materials for landing gears and they are selected in terms of strength, corrosion and fatigue [181]. The properties of the PMCs, MMCs and CMCs commonly used in aircraft are provided in Table 5.

## 6. Advanced Composites for Aircraft

Advanced composites have been implemented in aircraft structures due to their light weight, fatigue, and corrosion resistance [186]. For example, sensors that are mounted on lightweight carbon and glass fiber composites allow structural health monitoring (SHM) of aircraft, thereby to help in understanding the wave propagation as a result of different loading criteria [187].

### 6.1. Self-Healing Composites

Impact load causes composite materials to deteriorate. The impact damage starts as microscopic voids, which develop into profound microcracking and delamination in the structure, resulting in reduced structural integrity and premature failure. Previously, resin patches, injection, and heat plate techniques were used to fix these problems. However, those methods have several shortcomings, such as ineffectiveness for unseen damages, the requirement for damage monitoring, and inapplicability during construction activities. These factors restrict the uses of composites.

Materials that lessen damage or extend the lifespan and effectiveness of a damaged part, system, or device can increase their usefulness. One example of this sort of material is self-healing materials. The popularity of polymers and their composites as self-healing materials can be attributed to their increased molecular mobility [188]. Epoxy vinyl ester, bismaleimide tetrafuran (2MEP4F), raw polymer, cyclopentadiene derivatives, cyanate ester, and other composites including E-glass fiber-reinforced composites (FRCs) and carbon FRCs are a few self-healing materials that have recently been studied [188]. A hybrid multiscale polycarbonate composite with self-healing core-shell nanofibers at interfaces was also made via co-electrospinning. When interfacial damage, such as delamination, occurs in laminate composites, the core-shell is intended to self-heal [189].

To prevent delamination fracture of carbon fiber-reinforced plastic (CFRP) composites in aerospace applications, they were loaded with microcapsules with healing agents. A dicyclopentadiene-encapsulated microcapsule served as the healing agent and was combined with 20% by-weight epoxy resin. The specimens’ interlaminar fracture toughness was restored to 40% and 80% of their original values at room temperature and 80 °C, respectively. Utilizing a thermoplastic polymer matrix, thermally responsive polyurethane, and the Diels–Alder (DA) reaction, it was possible to repeatedly heal the delamination inside of a carbon fiber composite with 85% and 75% healing efficiency, respectively, throughout the first and second cycles [190]. By embedding CFRP with hollow glass fiber (HGF) within either glass fiber-reinforced plastic (GFRP) or carbon fiber-reinforced plastic (CFRP) and then infusing it with uncured resin, the self-healing feature was transferred to a laminate. Upon damage, a number of these fibers packed with resin burst, releasing the healing agent that has been stored there and initiating the healing process. The baseline laminate performance was roughly 89%, and this arrangement matched the undamaged condition by 97% [191].

Self-healing materials are typically used in aerostructures such as fuselages, wings, engines, cascades, and others as protective coatings or barriers. Hypersonic wings that are employed at temperatures exceeding 1600 °C typically have carbon/carbon composite nosecones, nozzles, and leading edges; however, at higher temperatures, oxidation occurs and lowers performance [192]. To avoid this, an oxidation-resistant outer layer is utilized to create an outer glass layer over an inner glass layer. Other oxidation-preventative barrier coatings include silicon carbide and silicon nitride coatings. Glass can flow into gaps to seal the covering against oxygen penetration when it melts at elevated temperatures [193]. As self-healing materials, silicon and boron-based particulate components in the carbon matrix are also employed. These substances react with oxygen to generate glass. Glass seeps into cracks, preventing oxygen from entering them. Additionally, the healing properties of the ethylene/methyl methacrylate (EMMA) copolymer were investigated for use in coatings for aerostructures. This polymer is incredibly self-healing and impact-resistant at high velocities [188]. Additionally, self-healing UV-responsive microcapsules have recently been researched for application in aeronautical coatings. They have a quickly degradable inner polymeric shell. When damage occurs, some of these microcapsules burst due to external pressure, and the remaining ones are destroyed by UV light, allowing the healing chemicals that were enclosed in them to be released and finally cure the cracks [194]. Self-healing materials based on polymer matrix composites are less expensive and simple to produce than ceramics and metals. Additionally, because self-healing ideas in metals and ceramics are still in their development, they are more complicated and challenging to put into practice.

### 6.2. Conductive Composites

Static charges build up on an airplane when it reaches a high altitude because of the interaction of the aircraft’s exterior with external environmental factors, which include air particles, ice, hail, dust, volcanic ash, and triboelectric charging. Parts and systems malfunction when a threshold value is surpassed due to explosions and broken radio transmission. Conductive composite systems made of a non-conductive polymer matrix supplemented with nanofiller or carbon-based nanocomposite/nanomaterials have addressed these problems [195]. With the reinforcement of carbon-based nanoparticles such as carbon black, carbon nanotubes (multi and single-walled), graphene, and epoxy/AgSWs coating, epoxy was envisioned as the most conventional matrix [196,197]. On the other hand, issues including non-uniform dispersion or higher loading have led to agglomerations and degradation in structural and electrical performance [198]. These materials also address the problems of lightning strikes and ice buildup in aircraft [199].

The epoxy resin-infused metal foams which were developed for the leading edge of the aircraft wings reduced wettability, insect adhesion, ice accretion, and particle wear to improve the flight performance, safety, and fuel efficiency of the aircraft. An innovative solution was explored in this work by infusing stainless steel composite metal foam (SS CMF) with a hydrophobic epoxy resin system. S-S CMF was made with 100% stainless steel using a powder metallurgy technique. The infused epoxy filled the macro- and microporosities, unique to SS CMF’s structure, creating a product with a density similar to that of aluminum [200]. Furthermore, to improve the electrical conductivity and flame-resistance properties, a carbon fiber-reinforced panel (CFRP) was impregnated with an epoxy resin using a combination of 0.5 wt% of carbon nanotubes (CNTs) and 5 wt% of Glycidyl-Polyhedral Oligomeric Silsesquioxanes (GPOSS) through a liquid infusion technique. The vibroacoustic tests confirmed an increment of the overall damping factor of the specimen due to the simultaneous incorporation of CNT and GPOSS fillers [201]. Moreover, resin reinforced with CNT for health-monitoring of aircraft primary structures was incorporated [202].

### 6.3. Resin-Infused Composites

To enhance flight safety, performance, and fuel efficiency of the aircraft, epoxy-resin-infused metal foams were designed for the leading edge of the aircraft wings. These foams reduced wettability, insect adhesion, ice accumulation, and particle wear. In this study, a novel approach was investigated by mixing a hydrophobic epoxy resin system with stainless steel composite metal foam (SS CMF). S-S CMF was created utilizing powder metallurgy and 100% stainless steel. The infused epoxy filled the macro- and microporosities, which were specific to the structure of SS CMF, producing a substance with an aluminum-like density [200].

Using a mixture of 0.5-weight percent carbon nanotubes (CNTs) and 5-weight percent glycidyl-polyhedral oligomeric siloxanes (GPOSS), an epoxy resin was infused into a carbon fiber-reinforced panel (CFRP) to further increase the electrical conductivity and flame-resistance capabilities. The simultaneous insertion of CNT and GPOSS fillers led to an increase in the specimen’s overall damping factor, according to the vibroacoustic measurements [201]. Additionally, this was also the case for resin reinforced with CNT for the basic structural health monitoring of aircraft [202].

### 6.4. Nanocomposites

Nanocomposites are also among the innovative materials used in composites and are distinguished from conventional composite materials by their superior mechanical qualities. CNTs, MWCNTs, and polymer-clay nanocomposites are among the types of nanocomposite materials that aim to address pre-existing issues in the aerospace industry [203,204,205]. To prevent the components of an aircraft system from degrading over time, it was discovered that molybdenum disilicate nanoparticles distributed in an aluminum matrix exhibited good wear resistance [206]. A graphene oxide (GO)-reinforced titanium nanopowder matrix technology was employed to achieve the high hardness that is a key goal in various structural aerospace components [207]. The aircraft industry’s use of nanocomposites in several subsystems, particularly due to the self-healing capabilities of nanocomposite polymers, illustrates the industry’s promising future [208]. Since the nanocomposite coating on jet engine turbine blades prevented grain formation even at extremely elevated temperatures, they were used as overlay coatings in several aerospace applications [209].

A PU matrix was embedded with conductive CNTs, and a slight increase in the CNT weight percentage resulted in a significant improvement in thermal diffusivity. Lower surface resistivity and flame-retardant qualities were discovered in the coating, making it perfect for aircraft applications [210]. By decreasing the capacity to absorb moisture, nano clay made it possible to postpone the breakdown of dielectric characteristics, assisting radomes in maintaining radar transparency. The performance of epoxy matrix in aircraft radomes is improved by the addition of nano clay particles [211]. Metal matrix composites (MMCs) were administered SiC and Al_2_O_3_ nanoparticle additions to see how they would affect the fatigue strength of aeronautical components [212]. The distribution of the grains and their size had a significant impact on the improved fatigue behavior, and it was found that increasing the percentage of nanoparticles increased the composite’s fatigue strength. A CNT-reinforced PP nanocomposite was used to create a micro air vehicle with a flapping-wing design inspired by biological structures [213]. In the realm of aircraft technology, nanocomposites have experienced great growth since the high-end applications required for the use of highly structural materials and nanocomposites appear to perform well [214].

## 7. Challenges and the Future Perspective

In recent times, the need for the development of MMCs for high-performance aircraft structures has rapidly increased. To overcome the existing limitations, the development of new advanced materials with different combinations of high strength, improved stiffness, and low density has become inevitable. To improve the applicability of MMCs in airframe construction and to withstand competition with the present polymer composites, significant investigations are required to evaluate their mechanical and structural performance. Moreover, this can be accomplished by increasing the strength-to-weight ratio or by reducing the absolute weight of the components. Furthermore, the challenging requirements of particular components such as low density and improved mechanical properties can be fulfilled by the proper selection of MMCs.

The development of safer materials for aircraft applications is crucial. This can be achieved by the usage of inflammable metallic composites with improved properties such as titanium-based alloys. The high-temperature resistance of Ti-based alloys can be improved through thermo-mechanical processing and controlling the phases by alloying. Furthermore, an increase in the usage of CMCs in commercial aircraft has been reported [176]. In the future, the major components of gas turbine engines would be replaced by CMCs except for a few components such as discs.

The major challenge for the commercial use of CMC is the high cost associated with the manufacturing process. It can be lowered by reducing the manufacturing time and increasing the usage volume. For the development of complex structures using CMCs, textile architectures will be required.

The control of the fretting wear, fatigue resistance, damage tolerance, and corrosion resistance of airframe materials affecting the maintenance, inspection, and repair costs is highly required. Such properties of metallic, ceramic, and polymer composites under different conditions need to be evaluated. This leads to the need for the development of new material with higher tribological and mechanical properties by various strategies and methods, including composition modification, microstructure refinement, control of impurities, coating, and employing improved fabrication techniques.

PMCs have a broad range of unanticipated applications. Nevertheless, it is equally important to make PMCs in a way that maximizes the value of the intrinsic qualities of these materials with their proposed applications. Due to the increasing demand for lightweight structures with efficient fuel consumption, PMCs will be used in large numbers in aerospace propulsion systems. Potential developments in PMCs for aerospace propulsion applications will involve multifaceted fiber textile architectures to deliver location-specific engineered properties. To serve the high-temperature application requirements, the development of PMCs will play a major role in aerospace propulsion systems.

Nevertheless, from weight reduction and improved structural performance of the materials, cost reduction through developed manufacturing techniques is also important. The costs associated with the manufacturing process would have a significant impact on the deployment of composite materials in the aircraft industry as the fabrication technique constitutes the largest portion of the cost of the airframe. Thus, great efforts are made to minimize production costs by introducing cost-effective and reliable techniques. Various investigations have been conducted to comprehend the manufacturing viability of MMCs [177,178]. In the last few decades, some of these processes have been utilized by the aircraft industry. However, lack of operator training, availability of homogeneous materials, process reliability, and cost offer a major hindrance for large-scale MMC applications.

A relatively pristine technology commonly known as additive manufacturing (AM) provides a significant potential to solve some of the problems in the context of the production of aerospace composites. It would lead to blending and merging the operations within one process, which at the moment is challenging to achieve with the existing composite production processes. It is important to note that AM will not resolve all the existing problems with conventional production processes, but it will positively transform the course of the development of composite materials.

## 8. Summary

The current review demonstrates that there has been significant growth in the development of new aircraft materials. The design specifications for aircraft structural materials demand that the materials should be damage tolerant and possess improved mechanical properties under various operating conditions. For several years, aluminum-based alloys have been employed as the primary materials due to their acquainted mechanical behavior, but their use at high temperatures is limited. Among all metallic composites, titanium has been proven to withstand high temperatures. However, some challenges limit the use of Mg-based alloys and Ti-based alloys in aircraft applications. In recent times, the use of polymer matrix composites has grown rapidly. This is due to their outstanding mechanical characteristics that include high stiffness and strength. The carbon fiber-reinforced polymer matrix composites have been explored extensively due to their high strength and lightweight but they are easily susceptible to stress concentration. The aircraft materials should possess suitable properties such as low density, and improved mechanical properties, and should be corrosion-resistant at high temperatures. Such properties of materials also depend on their manufacturing technique. The efficiency of the fabrication technique is determined by the type and volume of the fiber material or matrix used, as each material has distinct physical properties such as stiffness, tensile strength, melting point, and so on. In the future, significant investigations are required to discover new composites for structures by combining different variants and employing new manufacturing techniques.

## Figures and Tables

**Table 2:** Properties and applications of CMCs in aircraft.

Composites	Matrix	Reinforcement	Properties	Application	Ref.
ZrB_2_ or HfB_2_/SiC or Al_2_O_3_	ZrB_2_ or HfB_2_	SiC or Al_2_O_3_	-High oxidation resistance (2000 °C and above)	-Hypersonic flight (rocket propulsion and atmospheric re-entry)	[72]
ZrB_2_/SiC (Whisker or chopped fiber)	ZrB_2_	SiC chopped fiber or SiC whisker	-Good fracture toughness -High room-temperature strength -High-temperature strength	-High-temperature components	[73]
Oxide/oxide	Oxide	Oxide	-High performance -Reduced noise -Durability -Weight reduction	-Subsonic jet engines (exhaust mixer nozzle)	[74]
Glass-ceramic	Ceramic	Glass	-Lightweight -High temperature -Lightweight -Better performance -Reduced thrust-specific fuel consumption	-Aircraft compressor, combustor, and turbine	[75]
C/SiC	SiC	Carbon fiber	-Better tribological properties	-Aircraft brakes	[76,77]
C/SiC	SiC	Carbon fiber	-Bending strength -Fracture toughness	-Turbine blades	[78]
C/SiC	SiC	Carbon fiber	-Withstand temperatures up to 1200 °C	-Aircraft brakes	[79]
SiC	SiC	Carbon fiber	-Weight reduction -Improved retardation -Wear resistance -Improved carrier load and availability -Reduction in maintenance cost	-Aircraft brake (disks and rotors)	[80]
C/SiC	SiC	Carbon fiber	-Good thermo-erosive properties (up to 2000 °C) -High oxidation resistance -High strength-to-weight ratio	-Structural re-entry components -High-performance heat shields -Brake discs -Rocket nozzles -High-temperature heat exchanger tubes	[81]
C–SiC	SiC	Carbon fiber	-Average linear and mass erosion rate -Excellent resistance to thermo-oxidative erosion -Erosion resistance -High thermal conductivity -Good strength -Low CTE -Excellent thermal shock resistance	-Jet vanes	[82]
C/SiC	SiC	Carbon fiber	-Lightweight -Low density -High and stable coefficient of friction -High wear resistance	-Aircraft brake systems (brake pads and disks)	[83]
C/SiC	SiC	Carbon fiber	-Good thermal and mechanical properties -Higher Friction coefficients	-Aircraft brakes	[84]
SiC/SiC (CERASEPâA373)	SiC	Carbon fiber	-High fracture strength	-Engine combustor (rig and inner and outer liners)	[85]
SiC/C (SEPCARBINOXA262)	SiC		-Good specific strength	-Outer flaps (Rafale Fighter M88 engine A262)	[85]
SiC/Carbon fiber (C)	SiC		-Weight reduction (50%) as compared to superalloy flap (Inconel 718)	SNECMA M 88-2 engine (Flame-holders, engine flaps, and exhaust cones)	[86]

**Table 3:** Carbon fiber-reinforced carbon composites for aircraft applications.

Composites	Properties	Application	Reference
Carbon/Carbon composites	-Lightweight (40%) -Good thermal shock resistance -Good tribological properties -High heat capacity (2.5 steel) -High strength (2 steel)	Boeing 767–300 -Aircraft brakes (brake disc)	[87]
Carbon/Carbon composites	-Lightweight as compared to phenolic nozzle	Rocket nozzles (throat and exit cones)	[88]
C/C-SiC (SiC infiltrated C/C composites)	-Smaller size brake systems -High coefficients of friction -Higher transmitted braking power -Low wear rates (at temperatures above 1000 °C)	-Emergency brake systems	[89]
C/C-SiC (SiC infiltrated C/C composites)	-Microporous -High thermal shock resistance -Corrosion resistance -Good sealing agent for the pressurized pipes -Oxidation resistance at high temperatures	-Coated pipes	[90,91]

**Table 5:** Comparison of properties of PMCs, MMCs, and CMCs applied in aircraft [57,58,59,60,61,72,73,74,75,76,77,78,79,80,81,82,83,84,85,86,157,158,159,160,161,162,163,164,165,166,167,182,183,184,185].

Composite	PMCs	MMCs	CMCs
Microstructure	Long chain of molecules (fiber and matrices)	Predominantly metallic bond with a crystalline structure	Predominantly amorphous or crystalline structure
Mechanical	These are one of the lightest of the three composite materials and are found to have high specific strength and modulus. PMCs are brittle in nature.	These composites are ductile and have relatively high strength as well as high modulus compared to CMCs but are relatively heavier compared to PMCs.	These composites have very high strength and modulus compared to both PMCs and MMCs but are very brittle in nature.
Fracture toughness	Exhibit higher fracture toughness than CMCs	Have higher fracture toughness compared to PMCs and CMCs.	Exhibit lower fracture toughness among the three.
Fatigue	Have higher fatigue resistance compared to MMCs and CMCs	Exhibit better fatigue resistance compared to CMCs	Have low resistance under fatigue loading
Wear	Have higher wear resistance compared to MMCs	Exhibit lower resistance to wear compared to PMCs and CMCs	Exhibit higher wear resistance and hardness compared to PMCs and MMCs.
Creep resistance	High	High	Low
Density	Low	Medium	Medium
Operating Temperature	Up to 200 °C	Up to 800 °C	Up to 2000 °C
Application in Aircraft	Brakes, structures, wing boxes, fuselage, window frames, wing, rotors, brackets, boxes, bulkheads, fittings, airframe, blades, vertical fins, tail assemblies, food trays and arms	Landing gear, engines, aircraft wing, fuel tank (door part), and fans (F-16 fighter aircraft).	High-temperature components, subsonic jet engines (exhaust mixer nozzle), aircraft compressors, combustors, turbines, turbine blades, aircraft brakes (disks and rotors), structural re-entry components, high-performance heat shields, rocket nozzles, high-temperature heat exchanger tubes, jet vanes, aircraft engine combustor (rig and inner and outer liners), outer flaps (Rafale Fighter M88 engine A262), SNECMA M 882 engine, (Flame holders, engine flaps, and exhaust cones), Boeing 767–300 rocket nozzles (throat and exit cones), and coated pipes

## Data Availability

Data is available in the paper itself.
